# Convergence among Non-Sister Dendritic Branches: An Activity-Controlled Mean to Strengthen Network Connectivity

**DOI:** 10.1371/journal.pone.0003782

**Published:** 2008-11-21

**Authors:** Pablo Blinder, Joshua Cove, Maytal Foox, Danny Baranes

**Affiliations:** 1 Department of Life Sciences, Ben-Gurion University of the Negev, Beer Sheva, Israel; 2 Ariel University Center of Samaria, Ariel, Israel; Tel Aviv University, Israel

## Abstract

The manner by which axons distribute synaptic connections along dendrites remains a fundamental unresolved issue in neuronal development and physiology. We found *in vitro* and *in vivo* indications that dendrites determine the density, location and strength of their synaptic inputs by controlling the distance of their branches from those of their neighbors. Such control occurs through collective branch convergence, a behavior promoted by AMPA and NMDA glutamate receptor activity. At hubs of convergence sites, the incidence of axo-dendritic contacts as well as clustering levels, pre- and post-synaptic protein content and secretion capacity of synaptic connections are higher than found elsewhere. This coupling between synaptic distribution and the pattern of dendritic overlapping results in ‘Economical Small World Network’, a network configuration that enables single axons to innervate multiple and remote dendrites using short wiring lengths. Thus, activity-mediated regulation of the proximity among dendritic branches serves to pattern and strengthen neuronal connectivity.

## Introduction

Neurons present morphologically complex and diverse dendritic trees, the shapes of which play key roles in neuronal activity, as shown both theoretically [1], [2] and experimentally [3]–[5]. For example, the prediction that neurons bearing different dendritic morphologies differ in their responses to stimuli [6], [7] has been corroborated *in vitro* [8] and *in vivo* [9], [10]. Hence, the morphology of dendritic trees is a key factor in shaping synaptic activity in neuronal networks.

The physiological consequences of dendritic architecture cannot, however, be attributed solely to the structure of the individual tree. Rather, the spatial relationship of a dendritic tree with other dendrites must be considered since the activity of a given neuron is influenced by the distance of its dendritic branches from those of other neurons. When such distances are shorter than a few microns, the current produced by one active branch can spread through the extracellular matrix space to alter the membrane potential of an adjacent branch. Such non-synaptic neuronal communication, termed ephaptic coupling, is less versatile, and usually less specific, than communication at chemical synapses, yet may have functional consequences by causing activity synchronization [11], [12]. Hence, considering the geometrical map of dendro-dendritic proximity is essential for understanding the activity and morphogenesis of neuronal networks.

Is the distance between dendritic branches random, or is it determined by regulated processes? Support for the non-random model comes from observations that dendritic populations in different brain regions are organized into distinct configurations, some of which may favor ephaptic interactions. For example, Mauthner and Purkinje cells [13], as well as special types of hippocampal interneurons [14], tend to cluster their processes. The most intense closeness occurs in dendritic bundles, where dendrites present long contacts ranging from tens to hundreds of microns [15]. The high packing density in these bundles suggests that neighboring dendrites can influence each other through ephaptic interactions, thereby synchronizing their activities. Also, although in most brain regions dendritic trees overlap, in regions of sensory perception dendrites avoid each other, probably by secreting repulsive cues [10], [16]. Hence, the proximity among dendritic trees seems to be a complex, regulated process.

In this report, we find that dendritic branches of different dendritic trees converge in an activity-promoted fashion, resulting in clustering and strengthening of synaptic connections at the convergence sites. Such dendritic behavior led to formation of a specific network configuration, Economical Small World network, which broadens network connectivity by enabling single axons to innervate remote multiple dendrites in short wiring lengths. These results describe a novel activity-regulated structure-function relationship in neuronal networks. We, propose that this new link serves for inducing synaptic plasticity.

## Results

### Dendritic branches converge during culture development

**Figure 1:**
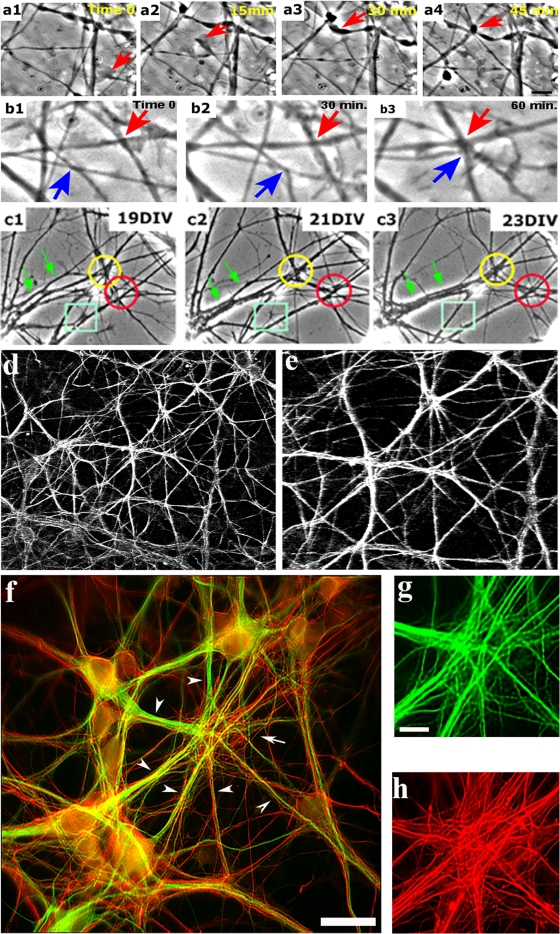
Mechanism and stability of neuronal cell process convergence. (a–b) time lapse series (phase contrast) showing process convergence through direct growth (a1–a4) and lateral movements (b1–b4). (c) time lapse showing two DCCs stable for three weeks (rings) and a DCCs dismantled at three weeks (rectangle). The image also shows a ‘zipper’-like mechanism of bundle formation and its stability for two weeks (green arrows). (d) phase contrast inverted image of a region with high frequency of convergence producing order in the network shape (e) higher magnification of (d) showing sites of multiple-process convergence. (f) convergence among 8–10 bundles of dendritic branches (green, anti-MAP2 antibody staining) and axons (red, anti-NFM antibody staining). The convergence site is shown in higher magnification in (g) and (h), where dendrites and axons, respectively, are shown. Note the high number of neurites that reach the convergence site and the absence of cell bodies. Scale bar: a–c, e, g, h – 10 µm; d – 30 µm: f – 20 µm.

Time lapse recordings of cultures at different ages revealed massive convergence of cell processes, either by the growth of processes towards preexisting contact sites between other processes (Figure 1a) or by the lateral movement of several processes towards a single area (Figure 1b). Such behavior resulted in the formation of clusters, several microns in width, comprising contact sites of multiple processes that remained stable over the course of several weeks (Figure 1c), a period corresponding to almost the entire life span of the cultures. In the cultures, cluster sites appeared sufficiently frequently so as to cause a ‘patchy’ network morphology (Figures 1d, 1e). A second type of process behavior involved the fasciculation of two or more processes over long distances, creating stable bundles (Figure 1b).

Efforts next focused on defining the composition of the converging processes, using specific antibodies directed against dendritic (microtubule–associated protein 2 (MAP2)) and axonal markers (Neurofilament (M NFM)). The clusters were thus shown to be comprised of numerous converging dendrites originating from many cells (Figure 1f, 1g), together with axonal (Figure 1h) bundles, but devoid of cell bodies. These convergence sites, termed dendro-dendritic contact clusters (DCCs), were first characterized in terms of dendritic behavior.

### DCC size and dendritic network aggregation increase with culture maturation and are promoted by synaptic activity

**Figure 2:**
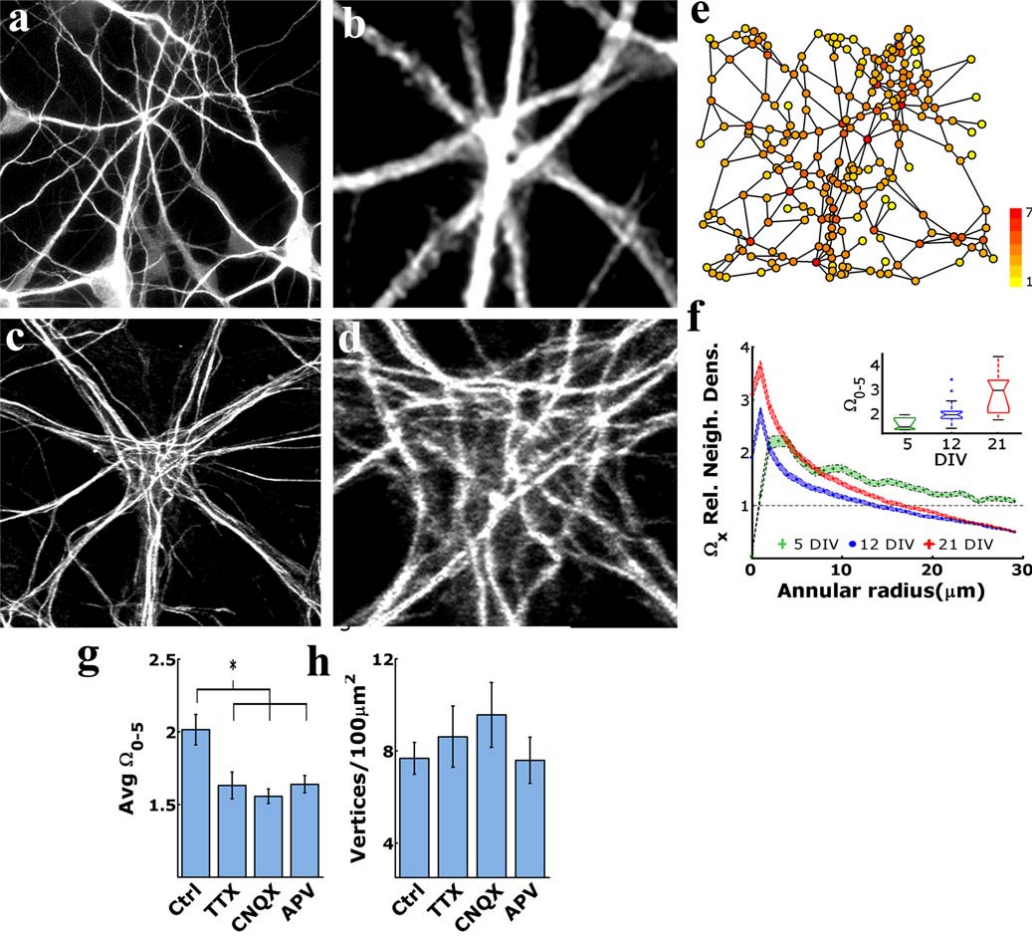
Dendritic networks aggregate with culture maturation in an activity-enhanced manner. During the first week in culture, DCCs were formed by convergence of few single dendritic branches (a) and had only few DCs (b, high magnification of (a)). In the second week in culture, DCCs formed from the convergence of many single and bundles of dendritic branches appeared (c). Such DCCs could include dozens of dendritic branches, forming a massive core dense with DCs (up to dozens of microns wide), containing no cell bodies (d, higher magnification of c).(e) A dendritic network (taken from (a)), represented as undirected graphs, obtained by manually positioning graph vertices at DCs (circles), and the dendritic segments connecting them as vertices (lines). Vertex coloring was assigned according to vertex degree, i.e. the number of adjacent vertices. (f) Ω_x_, the relative neighborhood density, depicted an aggregated pattern (values>1) in a DC distribution of short distances (inset). The network aggregation level is normalized by the global density (i.e. the number of DCs present in a given field divided by field area). Such normalization permits comparison between fields that bear different DC densities (18), where Ω_x_ was computed for three different densities as found in culture but for randomly distributed points. Aggregation at proximate distances (<5 µm) increased with culture maturation (green, blue and red boxes for 5, 12 and 21 DIV, respectively; mean±95% C.I.; p<0.01 by One-Way Anova; F = 8.36, df = 2 and n = 6,21 for 8 fields for 5,12 and 21 DIV, respectively; box lines represent lower quartile, median and upper quartile values, outliers (present only at 12DIV) are represented by outside whisker lines). (g) Inhibition of synaptic activity reduced the relative neighborhood density ( p<0.01 by One-Way Anova; F = 5.72, df = 3 and n = 21,9,9,9 networks from Control, TTX, CNQX and APV growth conditions, respectively; Tuckey post-hoc comparison, performed at an a = 0.05 significance level). (h) DC density in treated cultures did not significant differ from control cultures (p>0.05 by One-Way Anova). a–d - MAP2 antibody labeling. Scale bar: a,c - 10 µm; b, d - 3 µm.

In the first week of culture, DCCs contained few dendro-dendritic contacts (DCs), formed by a small number of single dendritic branches (Figures 2a, 2b). Over the next weeks, massive DCCs appeared, containing dozens to hundreds of DCs formed by convergence of dozens of single and bundles of dendritic branches into areas a few tens of microns in width (Figures 2c, 2d). Dendritic behavior was thus assessed by measuring the rate of DC aggregation. Based on the methodology described in Shefi et al. [17], images of dendritic networks were converted into graphs by defining DCs as nodes and the dendritic segments connecting them as edges (Figure 2e). The relative neighborhood density index, Ω_x_, an estimator of aggregation (or dispersion) [18], [19], was calculated by measuring the density of DCs found in concentric rings of defined radius (*x*) around each DC, then averaging over all DCs in the network. Comparing between networks was possible after normalizing by each networks average DC density. This calculation yields the level of ‘network aggregation’ (see Methods and legend to figure 2f for further explanation). As shown in the inset to Figure 2f, network aggregation levels increased with culture maturation. The average aggregation within 0–5 µm from a given DC reached 1.57±0.1 after 5 days in vitro (DIV), whereas after 12 and 21 DIV, this value increased to 2.01±0.1 (mean±95%, C.I.) and 2.74±0.29, respectively. The increase in network aggregation during culture development was maintained over the entire analyzed range of ring radii (0–30 µm, Figure 2f). Hence, during growth in culture, dendrites tend to accelerate their rates of convergence and contact with other dendrites.

Ω_0–5_ values were computed in 12 DIV cultures treated with different inhibitors of synaptic activity and found to be 25% (TTX), 29% (CNQX), and 22% (NMDA) below control, with the overall number of DCs being maintained (p>0.05, Figure 2h). Thus, DC aggregation is activity promoted.

### DCC formation is non-random and activity-enhanced

**Figure 3:**
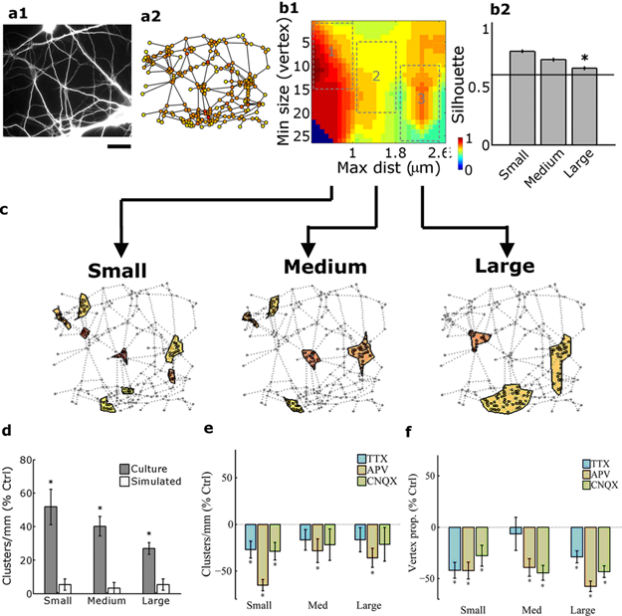
DCCs formation is non-random and is enhanced by synaptic activity. (a1) an example of a dendritic network (anti-MAP2 antibody staining) from which a DC connectivity graph was abstracted (a2). (b1) Clustering analysis of the graph based on maximal distance among the vertices and minimal size of the cluster. The colors represent the values of a Silhouette metric test. To facilitate further analysis, DCCs were grouped into three classes (small, medium and large, respectively reflected as 1,2 and 3 in b1, b2 and c1–c3). Only clusters bearing silhouette values above 0.6 were further considered for analysis. (d) The number of DCCs (normalized by total dendritic edge length in each field) is higher when compared to simulations of random contact-superposition (see methods and text for details) (p<0.01 by the Wilcoxon sing test, performed for each class separately; n = 33 and 10 fields for culture and simulation, respectively). (e) Synaptic inhibition has an overall decrimental effect on the normalized number of DCCs (p<0.05 for the medium cluster class by the Kruskal-Wallis test; n = 33 culture fields for control values and 11 fields for each treatment). When compared separately for each DCC class, all treatments lead to a decrease in the number of DCCs (p<0.001 by the Wilcoxon sign test). (f) Similarly, the proportion of the vertices involved in DCCs was decreased by synaptic activity inhibitors (p<0.05 for the medium and large cluster classes by the Kruskal-Wallis test; n = 33 culture fields for control values and 11 fields for each treatment). Here again, when compared separately, the proportion decreased in all but the small TTX-treated class (p<0.001 by the Wilcoxon sign test, same sample sizes). Scale bar: 12 µm.

The challenge in quantifying DCCs lays in defining their boundaries and deciding which DCs belong to a given cluster. Such decisions were made through ‘Hierarchical cluster analysis’, a widely used data-partitioning tool [20], [21] and subsequently confirmed by the ‘Silhouette’ metric approach [22], see also Supplemental Material S1 section 3. These criteria enabled us to discern between significant and non-significant data partitioning, assigning values of 1 to perfectly clustered data, 0 to ambiguous results and −1 to poorly partitioned data (Figure 3a1, a2, b1). For the sake of robustness and conservativeness, we opted to consider for further analysis only those DCCs with silhouette values greater than or equal to 0.6. This cut-off value, accepted for various types of biological data analysis [20], [23], [24], provided a DCC selection which best fitted our visual selection performed. Since DCC analysis depends on both the *minimal* number of DCs to be considered as a DCC and the *maximal* distance allowable for including a DC into a DCC, DCCs were subdivided as small, medium and large (Figures 3b,c), based on both threshold parameters (see Methods). Using these criteria, we compared DCC probability of appearance in 12 DIV cultures to that obtained in a computer simulation of randomly-distributed dendritic trees presenting a similar DC density as in the cultures (see Supplemental Material S1 section 1). Our comparison showed that the abundance of the three DCC classes (normalized per total dendritic length) in culture was higher than in the simulations, as shown in Figure 3d.

Next, the effects of inhibiting synaptic activity on: i) DCC density – the number of identified DCCs normalized to total dendritic length in each field and ii) DC clustering - the proportion of DCs that participate in DCCs, as a fraction of the total number of DCs in a given network, were considered. In general, DCC density and DC clustering decreased in response to all the activity inhibitors tested, with variations among the three DCC groups being observed (Figures 3e, 3f). APV was the strongest inhibitor of DCC density, being the only one to reduce that of all groups (Figure 3e). DC clustering showed more homogeneous sensitivity to the various antagonists used (Figure 3f). Thus, DCC formation is an activity-enhanced process.

### Clustering of pre-and postsynaptic proteins is enhanced at DCCs and is upregulated by synaptic activity

**Figure 4:**
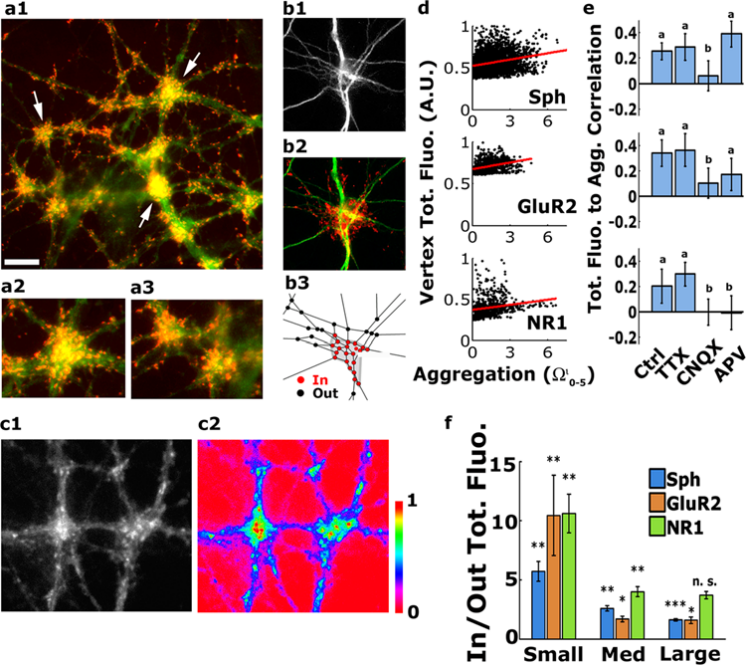
Activity-promoted synaptic clustering at DCCs. In all images (except c1 and c2), green = dendrites - anti-MAP2 antibody-staining; red = axonal varicosities - anti-synaptophysin antibody staining. (a1) Synaptophysin puncta aggregates (arrows) at DCCs. Note that the overall clumped synaptic organization in the field follows the DCC distribution. (a2) and (a3) Enlarged examples of DCCs and varicosity clusters, respectively, taken from (a1). (b1–b3) A representative field of the type used for quantitative analysis, where single dendritic processes (b1) and varicosities (b2) are clearly identified, and can be located within (‘In’) and out (‘Out’) of a DC cluster (red, silhouette value 0.751). (c1) Staining with anti-PSD-95 shows enrichment of postsynaptic densities at convergence sites. (c2) Color coding of c1 (scale bar: 0 = no fluorescence, 1 = maximal fluorescence). PSD-95 spots of the highest intensities are located at convergence sites. (d) A significant positive correlation between vertex-relative neighborhood density (Ω_0–5_) and vertex total fluorescence (see text for details) for both the presynaptic marker synaptophysin, and the postsynaptic markers, GluR2 and NR1 (0.25±0.03, 0.34±0.05 and 0.20±0.06 Pearson's correlation coefficient±95% C.I., n = 3130, 1113 and 789 vertices from 12, 4 and 4 fields for Sph, GluR2 and NR1, respectively). (e) CNQX abolished this correlation for all the markers; APV had a similar effect on NR1 distribution. (f) Quantification of the ratio (Inside/Outside DCCs) between the average intensity (total fluorescence (A.U.)/area) of the three synaptic markers. The synaptophysin density is always higher inside DCCs (p<0.01, 0.01 and 0.001 by the Wilcoxon rank test for the small, medium and large classes, respectively; n = 42,45 and 47 DCCs from 12 fields each, respectively). The distribution of post-synaptic GluR2 subunits of the AMPA receptor is also clumped around the three DCC classes (p<0.01, 0.05 and 0.05 by the Wilcoxon rank test for the small, medium and large classes, respectively; n = 10,17 and 18 DCCs from 4 fields each, respectively). The NR1 subunit of the NMDA receptor is clustered around small and medium but not near large DCCs (p<0.01 and 0.01 but p>0.05 by the Wilcoxon rank test for the small, medium and large classes, respectively; n = 9,9 and 12 DCCs from 4 fields each, respectively). .Scale bar: (a1) 20 µm, (a2–3) 15 µm and (b, c)10 µm.

To check whether dendritic convergence influences synaptic distribution, dendrites were co-labeled with antibodies to synaptophysin (Sph, a pre-synaptic marker) and to the GluR2 and NR1 subunits of AMPA and NMDA glutamate receptors and to post-synaptic density 95 protein (PSD-95) (post-synaptic markers). Antibody labeling clearly showed accumulation of pre- and postsynaptic proteins in DCCs to a higher level than found along dendritic segments located beyond the DCCs (Figure 4a–c). Synaptic clustering in the same type of culture was previously reported [25] but was not related to the manner of dendritic tree interaction. Correlation between DC aggregation and synaptic distribution was next assessed. The synaptic level at a DC was considered as the total fluorescence measured in a disk 5 µm in radius, centered at the DC. As reflected in Figure 4d, positive correlation between these two parameters was observed for both pre- and post-synaptic markers (0.25±0.03, 0.34±0.05 and 0.20±0.06, respectively). This correlation was diminished or completely abolished by treatment with CNQX or APV (Figure 4e). Thus, aggregation of DCs in the cultured network is associated with activity-enhanced synaptic clustering.

We next considered whether such clustering reflected an increase in synaptic density specifically at the DCCs. To measure that, DC-associated synaptic distribution at DCCs was computed as described in supplemental material S1 section 2. Briefly, the procedure involved the following steps: 1. Building a binary mask around the area covered by the dendritic network; 2. Computing the ‘In cluster’ mean intensity by defining DCC, as described above, creating a polygon with vertices around the most extreme DCs, summing the fluorescence intensity within the polygon and dividing it by the DCC's area. The average DCC intensity was then divided by the number of DCs found in the polygon. To keep track of the portions of the dendritic mask not included in DCCs, the polygon was then removed from the binary mask (computed in step 1); 3. Computing the ‘Out of cluster’ average was done by summing the total fluorescence in the remaining of the binary mask and dividing it by its area, and finally by the number of DCs which were not associated with any DCCs. The results showed Sph, NR1 and GluR2 to be consistently higher inside than outside a DCC, albeit to varying extents among the different DCC categories (Figure 4f). Hence, synaptic density is enhanced at DCCs compared to other areas along the dendrites.

### Enhanced pre-synaptic secretion occurs at DCCs

**Figure 5:**
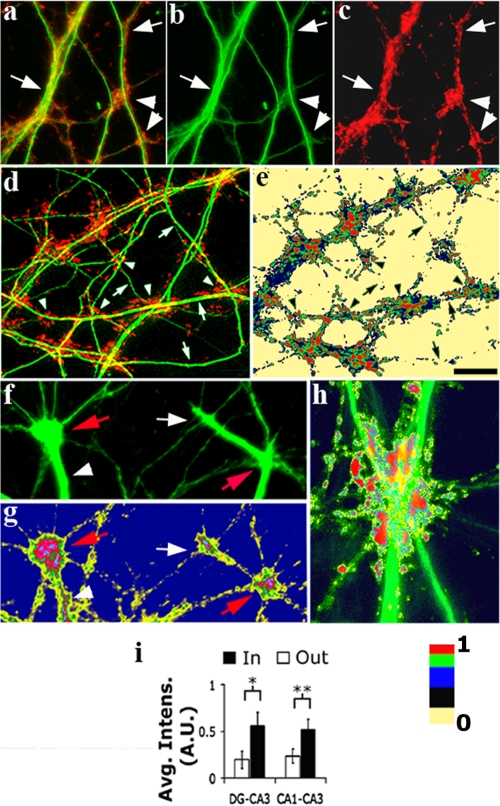
Presynaptic terminals at DCCs have stronger secretion capability than do terminals located elsewhere. In all panels, green represents dendrites (anti-MAP2 antibody labeling) and secreting terminals (FM1-43) are in red or color-coded according to the color scale at the bottom. (a–c) A region showing accumulation of presynaptic terminals at sites of dendritic bundling (arrows) and dendritic intersections (arrowheads). Note that the distribution of the terminals is determined by the above dendritic behavior. (d, e) Areas of dendritic convergence (d, arrowheads) are enriched with presynaptic terminals, as compared to non-converging areas (arrows). The level of FM1-43 uptake (induced by extracellular K^+^) by active terminals is higher in those located at convergence areas than those located elsewhere (e, color coding of d). (f, g) The level of FM1-43 secretion (induced by a second K^+^ application) by presynaptic terminals is stronger at bundling (arrowhead) intersection (white arrow) and convergence (red arrows) sites (g - color coding of f). (h) An example of FM1-43 secretion at a DCC showing that both the level of secretion and the size of the secreting terminals are higher than those of terminals far from the convergence area. (i) The FM1-43 average fluorescence is increased inside DCCs in DG-CA3 and CA1-CA3 cultures (p<0.001 by the t-test, n = 19 DCCs from 5 fields of each culture type. Color coding scale (right down): 0 = no fluorescence; 1 = maximal fluorescence. Scale bar (shown in e): a–g: 20 µm; h: 8 µm.

The question whether synapses clustered at DCC were of different strengths than other synapses was addressed by measuring presynaptic secretion levels using the synaptic vesicle recycling indicator, FM1-43. Distances between dendrites were tightly related to the distribution of FM1-43-positive puncta (Figures 5a–c), with terminals showing the highest endocytotic (Figures 5d, e) and exocytotic (Figures 5f, g) capacities being located at DCCs. Moreover, the size of individual terminals was higher at than out of DCCs (Figure 5h). We dissected out hippocampal sub-regions and measured secretion level from presynaptic sites within and outside DCCs in cultures of the mossy fiber and the Schaffer collateral pathways. Secretion level was measured by subtracting FM1-43 total fluorescence intensity in terminals after induction of release by K^+^ from that imaged prior to release (see full explanation in the method section). We found that terminals within DCCs had 2.8- (mossy fiber) and 2.2- (Schaffer Collateral) fold higher secretion level than terminals located outside DCCs, respectively (Figure 5i). These results indicate that synaptic connections are stronger at than outside DCCs.

### Convergence of dendrites into DCCs increases the number of contacts made with axons

**Figure 6:**
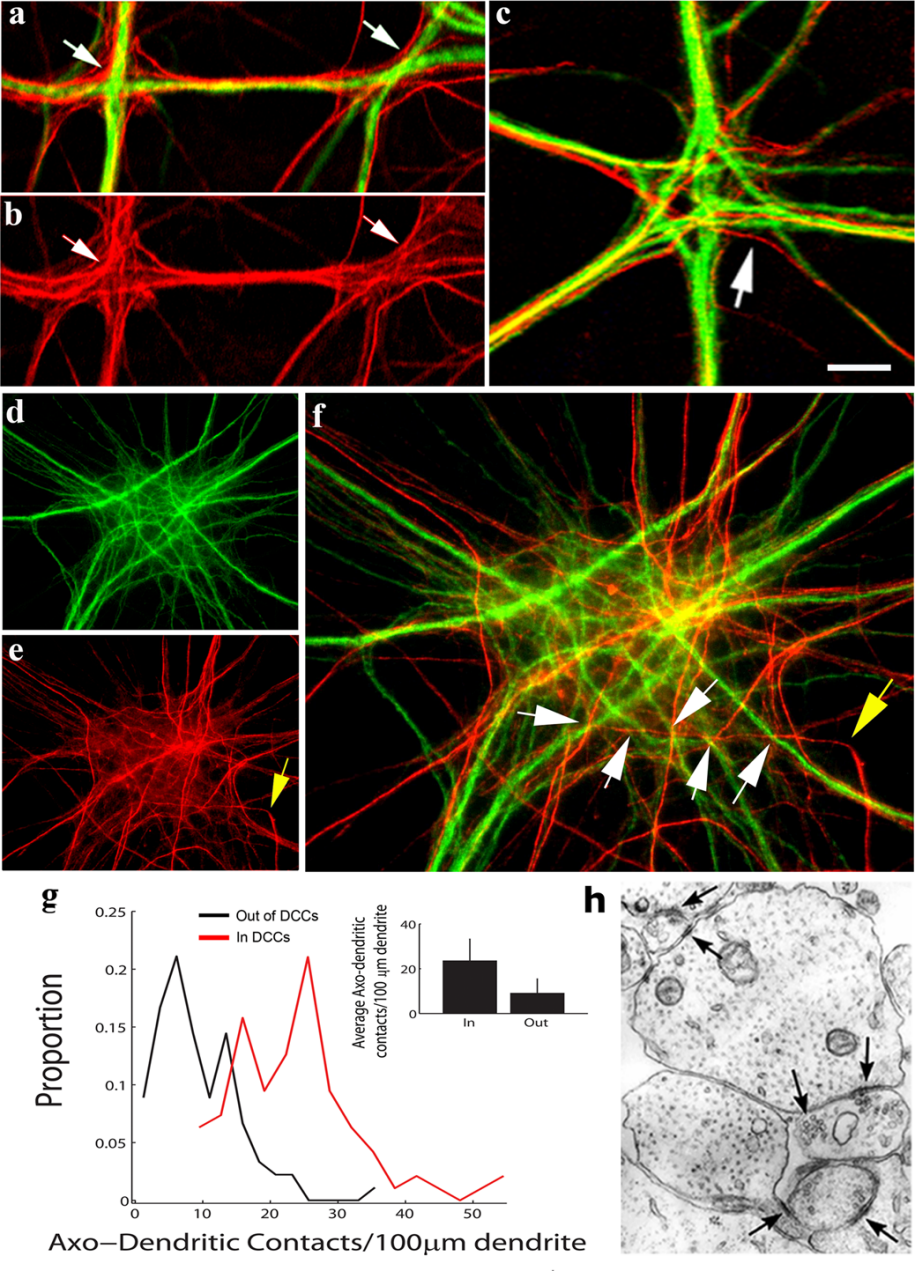
Axons increase the number of neurons they innervate by contacting multiple dendritic branches at DCCs. In all images, green represents anti-MAP2-labeled dendrites and red represents anti-NFM labeled axons. (A, B) Axons tend to fasciculate with dendrites but frequently when reaching a DC, they defaciculate, turn and switch dendrites (arrows). (C) An example of target switching at a small DCC (arrow). (D–F) A large DCC in which axons turn (an example pointed at by a yellow arrow in E), and form a complex mesh. The turning axons make contact with several different dendritic branches at relatively short lengths (white arrows in (f)). Yellow arrow is the same as in (e)). (g) Quantification showing a shift to the right in the number of axo-dendritic contacts per axonal length at (red) vs outside DCCs (black). (h) TEM micrograph of a longitudinal cross section in a DCC. Note individual presynaptic terminals forming synapses (arrows) with two different dendritic branches. Scale bar: a, b, f: 10 µm; c: 5 µm; d, e 15 µm; h: 0.5 µm.

We wondered if the high dendritic proximity at the center of DCCs could ease the way for axons to switch targets. Looking first at DCs, we found that axons fasciculating with a crossing dendrite often defasciculated near the DC site, turned and fasciculated with the other dendrite (Figures 6a, 6b). Similarly, axons reaching small DCCs through fasciculation with one of the converging dendrites often left the DCC to fasciculate on another dendritic branch, while at the DCC core, additional dendritic branches were contacted (Figure 6c). At large DCCs, axons form complex meshes (Figures 6d, e), within which the axons frequently turned and changed direction (Figure 6e). This behavior, combined with the high dendritic proximity at the center of the DCC, led to a situation where single axons switched targets and contacted many different dendritic branches in a relatively short length (Figure 6f). We noted that the density of intersections axons make with dendrites at DCCs is on average 2.5-fold higher than outside the DCCs (Figure 6g). Moreover, being close to several dendritic branches, many individual axonal terminals at DCCs made multiple synapses on different branches (Figure 6h). Hence, the presence of DCCs increases the number of neurons innervated by single axons in the cultured network.

### The structure of the dendritic network and its associated dendritic distribution assume an ‘Economic small world network’ configuration

**Figure 7:**
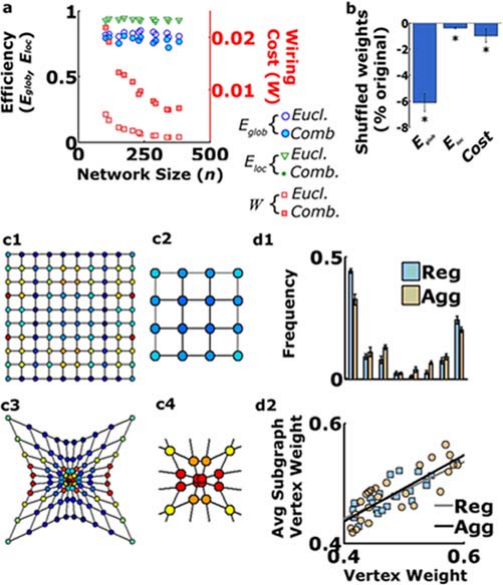
Convergence of dendrites and the concomitant patchy synaptic distribution yield an Economic Small-World Network topology. (a) Dendritic networks in two week old cultures show local and global efficiencies and wiring costs that fulfill the Economic Small-World requirements (i.e. having high Global and Local Efficiency while maintaining a low wiring cost, see text for details). Notice that both Eglob and Eloc do not scale with network size as opposed to Wiring Cost, pointing to a preserved organizational pattern. ESWNs also appear when synaptic weights (total synaptophysin fluorescence measured in a 2.5 µm diameter disk around each vertex) were included. (b) Disruption of the matching between dendritic network geometry and synaptic weights was achieved by shuffling the vertex synaptic weights while maintaining the same geometric organization of the network. This treatment always led to significant decreases in both local and global efficiencies, accompanied by an increase in wiring cost (p<0.0001, Wilcoxon sign-test for all metrics, n = 27 fields; each shuffling experiment was repeated 30 times and the average value used for comparison). (c) Synaptic clustering emerges as an optimal arrangement. The averaged synaptic vertex weight arrangement detected via a genetic algorithm (GA, see methods) that simultaneously maximized local and global efficiencies for regular (c1) and aggregated (c3) lattice-bearing but otherwise identical topologies. Notice the formation of a cluster of high synaptic weights matching the underlying aggregated dendritic vertex geometry (c4). Such an arrangement did not appear in regular lattices (c2). (d1) No significant differences were observed either in the averaged distribution of vertices weights or in the correlation between vertex weight and mean vertex weight of each vertex subgraph (n = 25 GA runs for each arrangement.

To check the role of dendritic convergence in shaping network architecture, we adopted the ‘Economical Small World Network (ESWN)’ analysis [26], [27]. This method estimates the overall efficiency of connectivity scheme in a network by providing information on its potential to propagate signals and its robustness (for a detailed explanation, see Supplemental Material S1 section 4a): 1. *global efficiency* – a value between 0 and 1 which reflects the speed of parallel information spread across entire networks and is inversely proportional to the average length of ‘shortcuts’ among distant network nodes. A value of 1 is assigned to an ideal (all-to-all) network, where all nodes are interconnected, and the length of each ‘shortcut’ between any two DCs is the shortest one. Departure from the all-to-all configuration yields lower values (closer to 0); 2. *local efficiency* – a value between 0 and 1 which is proportional to the number of local neighbors to which a given DC is connected [28]; and 3. *wiring cost* - a surrogate of the investment required for building a network of specific local and global efficiencies. In the case considered here, this value would correspond to a combination of dendritic length and the synaptic clustering (see Supplemental Material S1 section 4b). For a network to be considered as an efficient or economic SWN, its global and local efficiencies should lie in the proximity of 0.3 and 0.7, respectively, attained under a wiring cost below 0.3 [26], [27]. We found that all analyzed dendritic networks acquired an ESWN configuration (Figure 7a) while attaining higher efficiencies and lower wiring costs than other neural networks, such as the entire *C. elegans* somatosensory network or the cortico-cortical connections of both the macaque and cat [26], [27]. The efficiency values did not scale with network size (i.e. the number of DCs), whereas the cost value decreased with an increase in network size, pointing to a conserved organizational pattern (Figure 7a). In addition, the ESWN topological property of the cultured networks was proven to be activity-independent, as pharmacological inhibition of synaptic activity did not affect the measured values, relative to control conditions (not shown). Thus, the results show that dendritic trees in culture overlap to form a geometrical architecture that yields a high level of connectivity and ‘shortcuts’ among their DCs.

### The relation between dendritic network structure and synaptic distribution contribute to ESWN configuration

To assess whether the clustering of synaptic connections at DCCs is related to the network ESWNs configuration, two numerical experiments were performed. First, the efficiency analysis was redone after disrupting the clustering by randomly shuffling the synaptic weight assigned to each vertex. The reshuffling process was repeated 30 times for each network and an average parameter value was reported. Results from this numerical experiment design were best analyzed in a paired test, where it was possible to distinguish between pre- and post-shuffling effects on each individual trial. In all cases, disrupting the spatio-synaptic configuration *always* led to a decrease in global efficiency (6.09%±0.23; Figure 7b). Similarly, shuffling *always* led to a small, though significant, decrease in the local efficiency (0.4%±0.16). Wiring cost in the shuffled configurations was 0.95%±.1.73 lower. Thus, the results imply that dendritic networks self-organized into an efficient configuration by means of matching between the geometrical distribution of their contacts and that of synaptic sites.

In a complementary approach, an artificial scenario was created to demonstrate that matching between dendritic and synaptic distributions could emerge as a wiring principle for the optimization of network organization. A genetic algorithm (see Supplemental Material S1 section 5) was implemented to determine that configuration of vertex weights that simultaneously maximized both *E_glob_* and *E_loc_* of a lattice-like network under two different spatial configurations (i.e. regular and aggregated; Figures 7c1 and c3, respectively). The aggregated configuration presents the same topology as the regular case, only with the formation of a cluster of aggregated vertices at its center (Figure 7c3). The optimal configuration for the aggregated case (Figure 7c4), but not for the regular (Figure 7c2), averaged over 50 trials, resulted in a clustering of the highest vertex weights assigned to the vertices at the lattice core. Two additional clusters of vertices with high weights were formed at the lattice ridge, at a location where high weight vertices were also present in the regular case. The distribution of vertex weights did not differ between the two cases and showed bimodal-like behavior (Figure 7d1). The same relationship was, furthermore, observed between each vertex weight and the mean weight of its subgraph (i.e. the set of adjacent neighbors; Figure 7d2). Combined, these results demonstrate that matching between the distribution of dendrites and synapses in the culture contributes to the efficiency of the dendritic network SWN configuration.

### DCC-like structures exist in vivo

**Figure 8:**
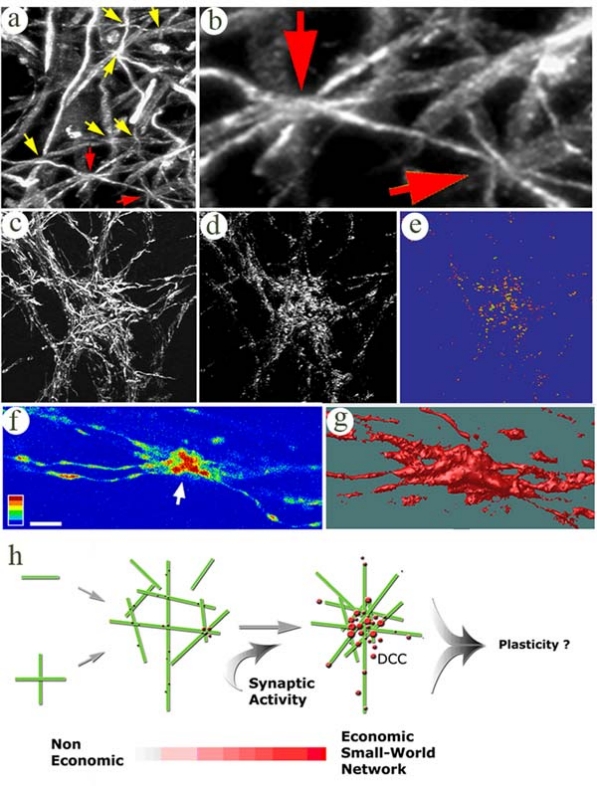
DCCs-like structures in the rat brain and the lobster stomatogastric system. (a) A confocal max projections of a 9 µm thick MAP2 labeled area in the Subtantia Nigra of adult rat brain having 8 sites of dendritic convergence. (b) higher magnification of two of these sites (red arrows in (a)) connected by a mutual dendrite. (c–e) 3D reconstruction of confocal sections of converging dendrites at another region of the Subtantia Nigra (c, MAP2) having clustered synaptic connections at the core of the convergence (d, synaptophysin) with the highest synaptophysin expression level (e). (f, g) Serotonin staining of the stomatogastric system of the lobster revealing neurite convergence where varicosity-like structures have the highest level of serotonin (f, arrow, single confocal section) and size (g, 3D reconstruction), relative to such structures elsewhere. h) Conceptual model of network structure consolidation. Green rods - dendrites, Red circles - synaptic clusters (darker colors refer to higher density and synaptic strength). For simplicity, axons are omitted from the model. When single or intersecting dendritic branches (green lines on the left) converge, DCC are formed. This process is promoted by synaptic activity. In the course of culture maturation and due to the formation of DCCs, the network becomes more aggregated. Synaptic clustering and strengthening becomes prominent at the DCCs. The geometric architecture of the dendritic network and the concomitant patchy synaptic distribution results in an Economic Small-World organization, a suitable scenario for minimal wiring length. The convergence phenomenon increases network connectivity and produces local enhancement in synaptic strength which may serve as a new mechanism of synaptic plasticity. Scale bar: a: 12 µm; b, f: 5 µm; c–e: 40 µm; g: 3 µm.

We then checked if DCCs exist *in vivo*. A first step considered whether DCCs are not merely a product of cell growth on a 2D surface. Confocal microscopy was employed to seek DCCs in adult rat brain. Due to their high dendritic density, areas like the hippocampus and the cortex were difficult to analyze, so we instead chose to focus on more disperse regions, like the Substantia Nigra. Because convergence in the brain occurs in three dimensions, our first approach was to collect sections from a 50 µm×50 µm area at a depth of 9 µm and merge them into a single image. Taking the average width of thin dendritic branches as 1 µm, the average distance between three converging dendrites at a 9 µm depth is about 3 µm, encompassing two synaptic connections, each arriving from a different dendritic branch. Using such values for dendro-dendritic contacts in 3D, we detected areas within the substantia nigra containing up to 8 convergence sites (equals to 3.2×10^5^ per mm^3^), each including 3–6 dendritic branches (Figures. 8a, 8b). On average, 33% of the dendritic segments located within the analysis area were involved in at least one convergence site and 12% were involved in more than one (Figure 8b, red arrows). DCC density in simulations of random 3D distribution of cylinders (with the same dimensions and density as in Figure 8a) was 15-times lower (not shown). We also found sites in the Substantia Nigra containing higher numbers of converging dendrites, associated with clusters of puncta expressing synaptophysin (Figures. 8c, 8d) at a higher level than those found beyond the convergence site (Figure 8e).

As DCCs were originally found in culture, we thought it logical to search for DCCs in more primitive neuronal systems than the brain, such as ganglions of the lobster stomatogastric system. Here, we found 6 convergence sites (3–4 processes each, Figure 8f) per 100 µm^3^ cube. Both the size and serotonin levels of synaptic puncta (Figures 8f, 8g) were higher at the convergence site that elsewhere.

Together, these results suggest the existence of convergence behavior *in vivo*, similar to that found in culture.

## Discussion

As summarized in Figure 8h, the present study shows that dendrites of cultured neurons aggregate over time by converging to form DCCs and bundles, and that this behavior is promoted by synaptic activity. At DCCs, dendritic branches are associated with higher synaptic density and strength than at other regions along the branches. It is also demonstrated that the coupling between synaptic distribution and the pattern of dendritic overlapping results in ‘Economical Small World Network’, enabling single axons to innervate multiple and remote dendrites using short wiring lengths. Hence, activity-enhanced bundling and convergence of dendritic branches induces local synaptic clustering and elevation in synaptic strength and, therefore, may serve as a new structural mechanism for plasticity in neuronal networks.

## 

### Proximity among dendrites is regulated by activity-dependent and –independent mechanisms

Our findings clearly show that dendritic convergence and the consequent dendritic network aggregation are regulated and directed biological processes that do not occur randomly. During three weeks of culture growth, a constant increase in network aggregation took place (Figure 2), whereas no increase was detected in simulated random networks (not shown). Indeed, had dendrites distributed randomly, a lower density of DCCs than that found in the cultures would have resulted (Figure 3d). Additional support for aggregation being a non-random event is its regulation by synaptic activity through the activation of both GluR2 and NR1 receptors (Figure 3e). Still, blocking activity only partially reduced DCC formation (Figure 3e), suggesting that other mechanisms are involved. It has been previously shown that neurite-neurite contacts acquire the capability to trap secretory trans-Golgi-derived vesicles [29]. It is, therefore, possible that contact at the intersection between two dendritic branches causes secretion of attractants that induce the convergence of additional branches to the intersection area, initiating DCC formation. Supporting this possibility, we found that long-term (overnight) exposure of neurons to FM1-43, so as to specifically label dendritic Golgi vesicles [30], resulted in enrichment at DCCs, as compared to non-converging regions of the dendritic network (Blinder and Baranes, unpublished). Therefore, DCC formation may be the response of dendritic branches to local gradients of attractants originating from DCs.

The role of synaptic activity in DCC formation may be underlined by a similar gradient-based mechanism, but for glutamate. If two crossing dendrites fasciculate with innervating axons, then synaptic density will be higher at and near the contact area, producing a gradient of glutamate. Single and bundled dendrites could then react to this gradient through their filiopodia [31] and grow toward the intersection vicinity to form a DCC.

Thus, in culture, dendrites exhibit activity-independent mechanisms of convergence, with activity up-regulating such behavior.

### The anatomical and physiological significance of dendritic convergence

#### I. The convergence of dendritic branches to DCC involves non-self recognition and shapes dendritic tree morphology

Studies describing dendritic morphology based on analysis of single dendritic trees often have led to the conclusion that dendritic ramification is random and that the growth directionality is unbiased toward specific targets [32], [33]. We present here a different explanation for dendritic tree morphogenesis, where the interactions of a tree with other trees are major players in the design of the final dendritic morphology. According to our model, the growth of dendritic branches is preferentially directed toward areas of high dendritic proximity to form bundles and DCCs. Thus, the development of particular dendritic tree architectures can be predicted by considering the distribution and density of DCs around the growing trees. By the same token, the morphology of entire networks of dendritic trees can be described by considering the number, location and size of their DCs, bundles and DCCs. Thus, studying dendritic proximity maps may enable us to proceed beyond the structure of individual dendritic arbors to that of full dendritic networks.

Interestingly, we did not detect DCCs that were formed by convergence among sister branches of a single dendritic tree. Rather, DCCs always included non-sister branches of several neighboring dendritic trees (Figures 1f and 2a). This ‘non-self’ manner by which dendrites associate indicates that sister branches undergo ‘self avoidance’, and that by preferentially converging with non-sister branches to form DCCs they highly increase the overlap among different dendritic trees.

#### II. The effect of DCCs on network connectivity

In relating to dendritic proximity by describing dendritic networks as graph of connections among DCs (Figure 2), we were able to show that in culture, such networks assemble into ESWN configurations (Figure 7). The main anatomical consideration of such a configuration is that a dendritic network exhibits ‘shortcuts’ that connect distant dendrites. Such an arrangement would have significant implications for axonal directionality and patterning, as many of the axons fasciculate with dendrites and follow their tracks (Figure 6). This means that if axons have access to ‘shortcuts’, their chances of innervating distant dendrites are increased, enhancing the connectivity of the entire network. This possibility also fulfills the ‘minimal wiring length’ principle guiding the wiring of many neuronal networks in the central nervous system [34]–[36].

However, the major contribution of the structural organization of dendritic networks to connectivity arises from the convergence of dendrites into DCCs. As reflected in the example presented in Figure 6f, axons reaching DCCs frequently contact many (up to several dozens, depending on the DCC size) of the converging dendrites. Due to the high proximity of dendrites, only a few microns of axonal growth and turning suffice for the axons to switch targets. Such an increase in target/axon ratio can be attributed to their minimal wiring length property, but also means that single cells would connect to a higher number of neurons in the network than would be the case in non-aggregated networks. The outcome of this wiring mechanism may be an all-to-all connectivity.

#### III. Relevance of dendritic proximity to network activity and plasticity

The convergence of dendrites, as well as its influence on synaptic distribution, may be of physiological relevance for several reasons: 1. As mentioned in the Introduction, the high dendritic proximity found at DCCs likely facilitates ephaptic coupling among the converging dendrites. 2. Clustering of synaptic connections at DCCs results in local reduction of inter-synaptic distances, as compared to those existing outside DCCs. It has previously been shown that clustered synapses exhibit firing properties of lower amplitude and higher frequency [37], as well as higher summation capability [38], as compared to non-clustered synapses. In this scenario, the high frequency of DCCs, especially in mature cultures, may play a significant role in activity patterning. 3. It is possible that at DCCs, due to the high density of synaptic connections and dendrites, glutamate spillover [39], [40] becomes an effective mechanism for activating multiple dendrites. 4. DCCs contribute to the pattern and total level of synaptic strength in the network, as synaptic connections at DCCs are richer in secretion capability (Figure 5) and in pre- and postsynaptic markers (Figure 4), as compared to those in non-converging dendrites.

In addition, taking into account that DCC formation is promoted by synaptic activity (Figure 3), these localized synaptic markers enrichments suggest that plasticity-related synaptic strengthening events transpire at these sites. This possibility is further supported by the fact that the formation of DCCs is partially mediated by synaptic activity through the NMDA receptor (Figure 3e), but the accompanied synaptic clustering and strengthening at the DCCs is promoted by AMPA receptors (Figure 3f). Such a distinction in glutamate receptors function is similar to that found in long-term potentiation of the mossy fiber pathway, where APMA receptors, and not NMDA underlie the plasticity induction [41], [42].

Combined, the above possibilities suggest that the map of dendritic proximities plays a role in patterning the activity of neuronal networks, as well as their plasticity. This hypothesis is supported by the findings that matching the geometrical architecture of dendritic networks and the corresponding synaptic distribution yields an ESWN configuration for the dendritic network (Figure 7a), a property which could enhance activity synchronization [43]–[46]. In addition, as a result of such matching, ESWN dendritic networks offer ‘shortcuts’ between distant parts of the network presenting the highest synaptic weight/length ratio (Figure 7). This idea is strengthened by the observation that when global and local efficiencies are maximized in a ESWN model, clustered distribution of synaptic connections is obtained (Figure 7c). A probable outcome of the existence of such ‘shortcuts’ is that dendrites following these shortened pathways should have a higher chance of interacting with synaptic clusters and, thereby, increase the density of inputs than had they chosen a ‘non-shortcut’ pathway. Moreover, as connections at DCCs are of higher strength than elsewhere, ‘shortcut’ pathways may increase the chance of dendrites to be synaptically activated. Hence, dendritic convergence promotes synaptic weight-based ESWN organization, thereby patterning and increasing the strength of connectivity and activity in neuronal networks.

We conclude that the proximity among dendritic branches of neighboring neurons is a functional structural entity. Being up-regulated by synaptic activity and associated with enrichment in synaptic density and strength, dendritic proximity affects the conversion of synaptic information into a map of synaptic connections and synaptic strength distributions. Accordingly, when neuronal network activity increases, the network architecture becomes more aggregated through DCC and bundle formation, leading to an increase in synaptic clustering and strength. This structure-mediated, activity-dependent synaptic strengthening may serve as a novel structural-based mechanism of plasticity.

## Methods

### Cell and tissue culture and treatment

CA3 and dentate gyrus from brains of postnatal rats were treated with trypsin (Sigma, type XI), triturated, and the released cells were plated (2×10^5^ cells/ml for 2D and 3×10^8^/ml for 3D cultures) onto glass cover slips or coral skeletons coated with poly-D-lysine (Sigma, 20 µg/ml) and laminin (Collaborative Research, 10 µg/ml), in a culture medium containing 10% serum, as described previously [47]. Cultures were maintained in a 37°C incubator in a 5% CO_2_ atmosphere in the presence or absence of CNQX (10 µM), APV (50 µM) or TTX (1 µM).

### Immunocytochemistry

For immunocytochemical study, cells were labeled as described previously [48]. Briefly, cells were fixed for 10 min at room temperature with 4% paraformaldehyde, permeabilized with 0.25% Triton X-100, and blocked with 3% normal goat serum. The cells were then incubated overnight at 4°C with anti-MAP2 (1 µg/ml) (monoclonal, Sigma, Oakville, Ontario, Canada); anti-NFM, anti-GluR2, anti-NR1, anti-PSD-95 (0.5 µg/ml) (polyclonal, Chemicon, Temecula, CA, USA) or anti-synaptophysin (0.5 µg/ml) (polyclonal, DAKO, Mississauga, Ontario, Canada) antibodies. Immunolabeling was visualized with secondary antibodies conjugated to Alexa-488 or Cy3 (2 µg/ml) (Molecular Probes, Eugene, OR, USA).

### Light microscopy

Images were obtained using Axiovert 200 M microscopes with Plan-Neofluar 20×/0.5 and Plan-Apochrome 63×/1.4 objectives, equipped with 12-MHz CCD cameras (SensiCam, PCO, Kolheim, Germany). Acquisition and analysis were performed under Metamorph v6.3 (Molecular Devices, USA) or via custom-made MATLAB 7.0 software (Matworks, Massachusetts MA, USA). Figures were processed using PhotoShop 7.0 (Adobe Systems, San Jose, USA).

### Measurement of pre-synaptic secretion

Cultures maintained for 14–16 DIV were exposed for 30 sec to 15 µM of the synaptic vesicle-recycling marker, FM1-43 (Molecular Probes, Carlsbad, USA), in HEPES/Tyrode's buffer and 90 mM K^+^. After a 3 min wash, ‘uptake’ images from two to four randomly selected fields were acquired under non-saturating pixel value conditions. FM1-43 was then secreted by application of 90 mM K^+^, followed by a 3 min wash with Tyrode's buffer and the previously acquired fields were imaged again under the exactly the same imaging conditions (i. e. illumination setting, exposure time and binning); this defined the ‘release’ set of images. Finally, vesicle-recycling activity was estimated by computing the ‘net release’ image, obtained by subtracting the ‘release’ from the ‘uptake’ images.

### Transfection of cells

Cultures were transfected between 5–10 DIV, as described previously [49]. In brief, cells were washed and incubated for 45 min at 37°C with MEM containing 0.5% glucose. Each coverslip was then incubated for 30–40 min at 37°C with 80 µl of DNA solution until a heavy precipitate formed (DNA solution: 5 µg DNA (plasmid pIRES2-EGFP, Clontech), 250 µl of 250 mM CaCl_2_ and 250 µl of BBS (in mM: NaCl 280, Na_2_HPO_4_ 1.5, BES 50, pH 7.1)). Finally, cells were washed twice with HBS and twice with MEM, and then returned to their original growth medium. Cells were imaged 5–8 days after transfection.

### Transmission electron microscopy

Primary fixation was done with modified Karnovsky's fixative (2.5% glutaraldehyde, 2% paraformaldehyde in 0.1 M cacodylate buffer (pH 7.2) for 30 min at 37°C. The samples were post-fixed with 1% osmium tetroxide at 4°C for 30 min. Samples were dehydrated in graded ethanol series and propylene oxide, followed by a gradual embedding in Araldite 502 (Electron Microscopy Sciences, Fort Washington PA, USA). Sections were cut using Leica Ultracut UCT microtome (Leica Microsystems, Nussloch, Germany), contrasted by Uranyl acetate and lead citrate and observed in Jeol JEM-1230 TEM (JEOL LTD, Tokyo, Japan) at 80 kV. Electron micrographs were taken using TemCam-F214 (Tietz Video & Image Processing Systems (TVIPS) Gauting, Germany.

### Graphic representation of dendritic networks based on DCs

Images of dendritic networks (MAP2 staining) were manually converted into graphs (Figure 3a1,2) by implementing a graph deduction paradigm similar to the one used by Shefi *et al.* [17], where vertices were marked mainly at dendro-dendritic intersections, cell bodies, bifurcations, or growth cones (Figure 3a1,2). Dendritic segments connecting such vertices were considered as edges (Figure 3a2). Graphic reconstruction was performed via a custom MATLAB graphical user interface developed for this purpose (available for academic use at www.bgu.ac.il/~blinderp/public_html/software.html). The outcome of the graph abstraction process is a fully connected planar graph *G (V, E)*, defined by the set *V* of its *n* vertices and *E*, the set of edges. Convenient representation and handling of such graphs involves the *adjacency* matrix, *A*, where each of its *a_ij_* entries specifies the existence of a link (edge) between vertices *i* and *j*:
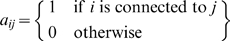
(1)


The graph *G* is planar as any edge cross is considered a vertex and is made symmetric, (i.e. *a_ij_* = *a_ji_*), as no information regarding the direction of signal propagation *along* each edge (dendritic segment) is available from the real network.

### Measuring network aggregation levels

The level of aggregation of the dendritic networks was estimated by implementing the relative neighborhood index (Ω_x_) [18], [19]. For a given system with discrete elements in space, this analysis measures the aggregation (or dispersion) based on the change in annular density (d_x_) over a specific spatial range of interest. Here, d_x_ is the number of DCs found in a ring divided by its area (a_x_). Each ring is defined by x and x+Δ x, the inner and outer radii respectively. For each DC, a series of d_x_ values were computed by placing a set of concentric rings. In the current analysis, x ranged from 0 to 30 µm with Δx = 1 µm. Averaging this metric across the n DCs in a given network yields the global annular density D_x_:
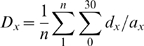



Finally, to compare between different networks, D_x_ is normalized by the absolute DC density d′; resulting in the relative neighborhood index, Ω_x_ = D_x_/d′. The d′ metric consists of the number of DCs divided by the area of the network. Since the network is arbitrary defined by the extent of the field of view (see fig. 3a for example), the area of the network was measured in a polygon defined by connecting the outer most DCs. By definition [18], normalized values above 1 indicate a clustered spatial distribution, while values below 1 point toward spatial over-dispersion (i.e. more empty spaces).". It follows from this that –for a finite system–, if clustering is identified at a specific range, one should expect a complementary over-dispersion at a longer range.

### Computing vertex synaptic weights

Two complementary measurements of vertex synaptic weight were performed and implemented, depending on the specific analysis. While focusing on DCs *per se* (as previously described for the aggregation pattern), synaptic distribution was estimated as the total fluorescence measured in a disk of 5 µm radius. This approach is referred to here as the disk method. In turn, while considering the collective properties of groups of contacts (i.e. DCCs), vertex synaptic weight was computed by measuring total fluorescence in the area of a polygon (defined by the most extreme vertices of the DCC) intersected with an appropriate dendritic mask (Supplemental Material S1 section 2) and then dividing this value by the number of vertices in the DCC.

### Approval of animal usage for experimentation

The usage of rats in this work was approved by the Ben Gurion University Committee for the Ethical Care and Use of Animal in experiments (Authorization number: IL-17-2-2005).

## Supporting Information

Material S1 (2.58 MB DOC)
