# Supplementary material for: Convergence among Non-Sister Dendritic Branches: An Activity-Controlled Mean to Strengthen Network Connectivity
Source: PLoS One. 2008 Nov 21;3(11):e3782. doi: 10.1371/journal.pone.0003782 (PMC2582457; doi:10.1371/journal.pone.0003782)
Supplement: Material S1 — (2.58 MB DOC) [file pone.0003782.s001.doc]

# Supplemental Material S1

# Section 1

# Simulation of random dendritic network formation


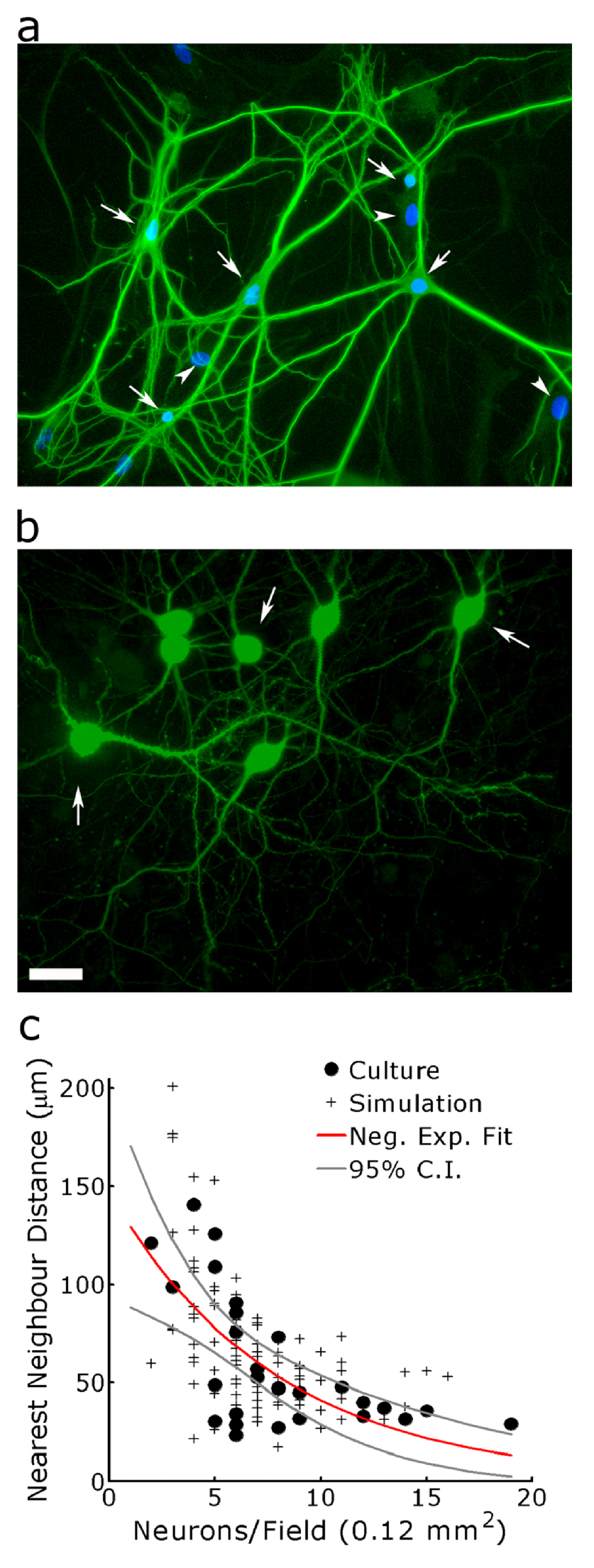
‘Pseudo-random’ networks were constructed from a dataset of images of single GFP expressing cells (n=26, see Methods). The images where randomly sampled with replacement, rotated and positioned at random to form a larger new composite image. To allow for suitable comparison, cell density and spatial distribution were selected to match the observed patterns (). A suitable metric that encapsulates these two factors (density and spatial arrangement) is the ‘nearest neighbor distance’ between cell nuclei. Thus, we tuned the simulation parameters to ensure that no significant differences where appreciable (C). By doing so, the elemental morphological features of single cells and their dendritic arbors remain unchanged, while the formation of contact sites between neighboring cells resulted from a random process (S1.1). Following construction of the composite image, we applied our manual graph abstraction procedure to generate random graphs suitable for further analysis and comparison to observed cultured networks.

**Reference**

S1.1 [Beletti ME](http://www.ncbi.nlm.nih.gov/sites/entrez?Db=pubmed&Cmd=Search&Term="Beletti ME"%5BAuthor%5D&itool=EntrezSystem2.PEntrez.Pubmed.Pubmed_ResultsPanel.Pubmed_DiscoveryPanel.Pubmed_RVAbstractPlus), [Costa Lda F](http://www.ncbi.nlm.nih.gov/sites/entrez?Db=pubmed&Cmd=Search&Term="Costa Lda F"%5BAuthor%5D&itool=EntrezSystem2.PEntrez.Pubmed.Pubmed_ResultsPanel.Pubmed_DiscoveryPanel.Pubmed_RVAbstractPlus), [Viana MP](http://www.ncbi.nlm.nih.gov/sites/entrez?Db=pubmed&Cmd=Search&Term="Viana MP"%5BAuthor%5D&itool=EntrezSystem2.PEntrez.Pubmed.Pubmed_ResultsPanel.Pubmed_DiscoveryPanel.Pubmed_RVAbstractPlus). (2004) [Biotech Histochem.](javascript:AL_get(this, 'jour', 'Biotech Histochem.');) A computational approach to characterization of bovine sperm chromatin alterations. 79(1):17-23.

###

# Section 2

### Building dendritic masks for synaptic weight estimation at DCCs

For quantification of the dendritic network architecture, a dilated mask of MAP2-labeled dendrites was constructed based on the geometrical properties of the network edges (Section 2 figure ). In determining the dilation factor, we followed Kruger and coworker’s definition of ‘in dendrite’ space, i.e. a space <~0.35μm from the border of MAP2 staining (S2.1). We reconstructed portions of the dendritic image and added them up to form the final mask by selecting combinations of the peak values from the distributions of both the edge orientation and length present in each image (Section 2 figure c).

Once the mask has been constructed, it is possible to compute the synaptic weight, i.e. the total fluorescence of a specific synaptic marker, divided by the area of specific portions of the dendritic mask. For example, once a DCC is successfully identified (see Methods), the average synaptic weight of each of its elements (DCs) consists of the total fluorescence measured in the area of the cluster that coincides with the dendritic mask, divided by the number of vertices in the cluster. This is, in general, an underestimation of the synaptic weight as the area of the cluster is larger than the area confined by the dendritic mask. This occurs due to the presence of ‘out-dendrite space’ between edges. Since this space is confined inside the cluster perimeter -as defined by connecting the most extreme vertices members of the cluster- it is included in the clusters area, even though it did not include a dendritic segment. Thus, one would expect to measure only background fluorescence signal leading to lower total fluorescence values for the entire cluster area.

## Reference

## S2.1. [***Krueger SR***](http://www.ncbi.nlm.nih.gov/sites/entrez?Db=pubmed&Cmd=Search&Term="Krueger SR"%5BAuthor%5D&itool=EntrezSystem2.PEntrez.Pubmed.Pubmed_ResultsPanel.Pubmed_DiscoveryPanel.Pubmed_RVAbstractPlus), [***Kolar A***](http://www.ncbi.nlm.nih.gov/sites/entrez?Db=pubmed&Cmd=Search&Term="Kolar A"%5BAuthor%5D&itool=EntrezSystem2.PEntrez.Pubmed.Pubmed_ResultsPanel.Pubmed_DiscoveryPanel.Pubmed_RVAbstractPlus), [***Fitzsimonds RM***](http://www.ncbi.nlm.nih.gov/sites/entrez?Db=pubmed&Cmd=Search&Term="Fitzsimonds RM"%5BAuthor%5D&itool=EntrezSystem2.PEntrez.Pubmed.Pubmed_ResultsPanel.Pubmed_DiscoveryPanel.Pubmed_RVAbstractPlus). (2003) The presynaptic release apparatus is functional in the absence of dendritic contact and highly mobile within isolated axons. [***Neuron.***](javascript:AL_get(this, 'jour', 'Neuron.');) ***40(5):945-57.***

##
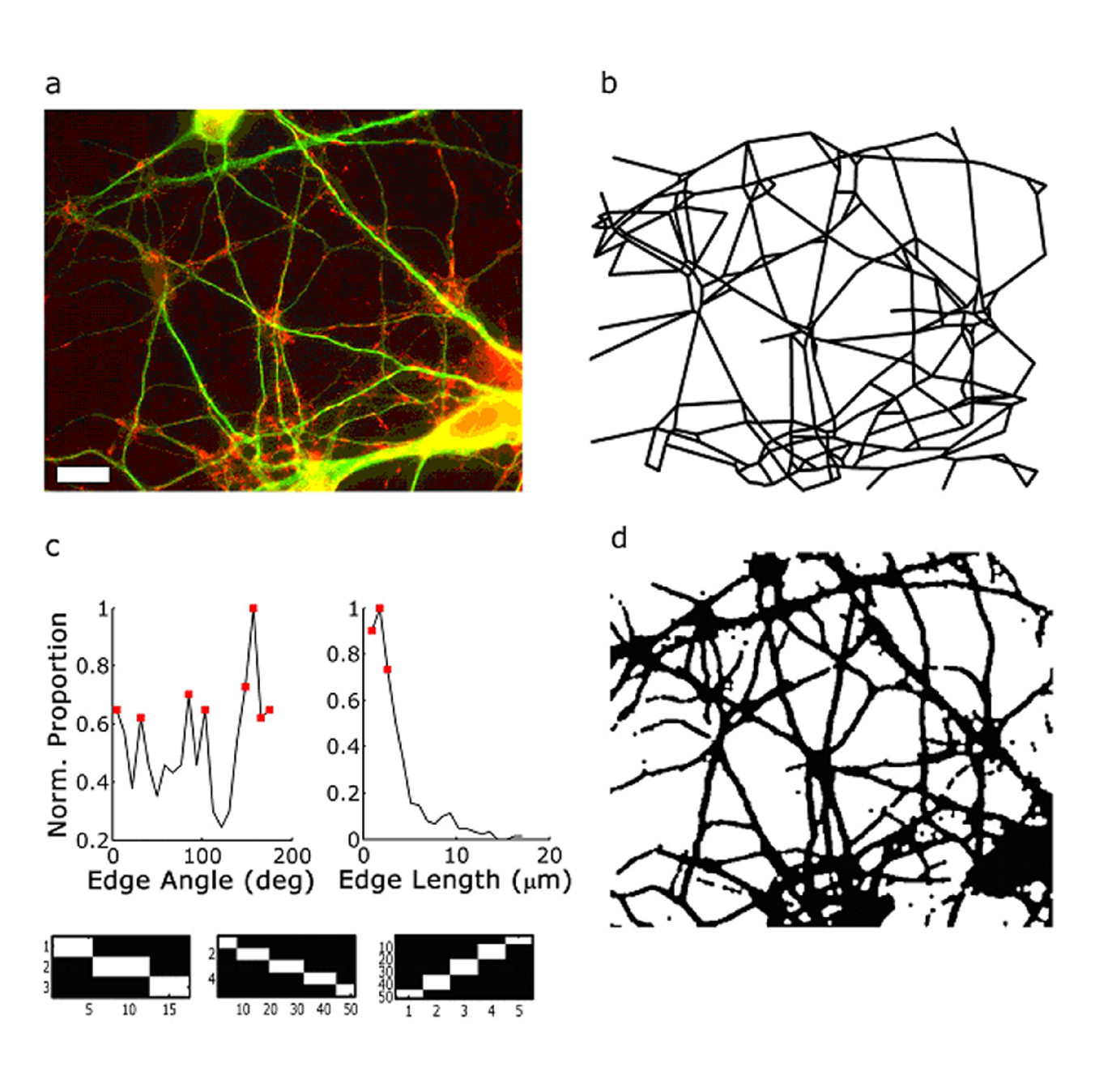


##

**Section 2 figure 1. Building a mask for the estimation of synaptic distribution across the dendritic network.** (a) A composite image of a dendritic network (green, MAP2-positive) and synaptic vesicle aggregates (red, synaptophysin-positive puncta). (b) The abstracted graph from the dendritic network in (a) (showing edges only). (c) Edge angle and length distribution of (b). Red squares represent selected values. For each combination of values from the two distributions, target objects were constructed (c bottom, showing only three possible combinations; notice the different scales). These objects are iteratively implemented as morphological filters to build a mask combined from the result of each filtered image. A dilation process is implemented as the final step in constructing the dendritic mask (d). Scale bar, 10μm

# Section S3

### SP 3 - Hierarchical clustering analysis and its validation via the Silhouette method

Here we applied Hierarchical clustering (21), an approach that does not require *a priori* knowledge of the expected number of clusters, to group graph vertices for the identification of DCCs. Briefly, for each vertex, *i,* the Euclidian distance, *d_ij_*, to the remaining vertices in the network, *j*, is computed. Next, a dendrogram is constructed based on *d_ij_*. As the similarity between two vertices increases, the number of branching points in the dendrogram separating them decreases. The final number of identified clusters thus depends on two threshold parameters: 1. *d_max_*,, the maximal distance acceptable for including a new member to a forming cluster and 2. *n_min_*, the minimal number of vertices on each of the cut-off products that will ultimately be considered as a DCC. To avoid any bias in the analysis, we performed the clustering process under a wide range of parameter values. The *d_max_* parameter ranged from 0.5 to 4 μm, while the *n_min_* parameter ranged from 5 to 50 vertices per DCC. However, for simplicity of data presentation and analysis, three DCC groups (small, medium and large (Figure 3b-c)) were defined, based on both threshold parameters.

One crucial issue in the field of cluster analysis is the estimation of the quality of data partitioning. Although several methods are suitable for this task, the *silhouette* metric provides an intuitive and computationally effective tool for this purpose (22, 23). Furthermore, this approach has already been employed in the study of neuronal morphology (20) as well as in large scale genomic micro-array data analysis (24). Briefly, this metric is derived from the average similarity value for each point in a given cluster and is a measure of how similar that point is to other points in its own cluster, as compared to points in other clusters and is described by:

$S\left(i\right)=\frac{b\left(i\right)-a\left(i\right)}{\max\left\{a\left(i\right),b\left(i\right)\right\}}$ (*Eq. S3.1)*

where *S(i)* is the silhouette value of the *i^th^* element in a given cluster, *a(i)* is the average dissimilarity of the *i^th^* element to all other element in the same cluster and *b(i)* is the minimum of average dissimilarity of the *i^th^* element to all objects in the closest cluster (22). Since the Euclidean distance is used both for the clustering and silhouette analyses, dissimilarity *a(i)* is, therefore, the distance of the *i^th^* element to the center of its cluster. It thus follows from *Eq. 2* that silhouette values range from -1 to +1. A value close to 1 describes a well-clustered result, where each element is associated to an appropriate cluster. A value close to 0 represents ambiguity as *a(i)* is close to *b(i)* and will cast doubt on the assignment of this element to the cluster. Values closer to -1 represent a misclassified clustering, since *a(i)* is bigger than *b(i)*, indicating that the *i^th^* element will be better assigned to another (closer) cluster.

# Section S4a

### Analysis of wiring efficiency (Economic Small-World networks) in dendritic networks

The formulation of *efficiency,* *e_ij_*, is based on the assumption that information travels more efficiently between two connected vertices the closer they are to each other, meaning that *e_ij_* is proportional to the inverse of the length of the shortest path between the two vertices. Hence, the efficiency of a network (global efficiency) is defined as the average of efficiencies of all pairs of vertices. The average *efficiency* of graph *G* is then defined as:

$E\left(G\right)=\frac{\sum_{i\neq j\in G} e_{ij}}{N\left(N-1\right)}=\frac{\sum_{i\neq j\in G} \frac{1}{l_{ij}}}{N\left(N-1\right)}$ *(Eq. S4a.1)*

To compare efficiencies between different networks, global efficiency has to be normalized using the normalizing factor, *E(G^ideal^).* This factor reflects the ideal efficiency of a network with the full N(N-1) set of possible connections, where information flow is maximized, with every short path between two vertices equaling the Euclidean distance between them. Thus, *E_glob_*, as defined in Eq. *S4a.2*, is of a value 0≤ *E_glob_(G)* ≤1. *E_glob_(G)* equals 0 when *G* is completely unconnected and equals 1 when *G* is a complete graph (*G^ideal^*, as defined above).

$E_{glob}=\frac{E\left(G\right)}{E\left(G^{ideal}\right)}$ *(Eq. S4a.2)*

The averaged local efficiency metric, *E_loc_*, is based on a generalization of the former parameter for a subset of subgraphs of *G*. This subset consists of all *G_i_* subgraphs, where *G_i_* is the subgraph made by the adjacent *k_i_* neighbors of node *i* (*i∉G_i_*). This parameter estimates local connectivity and network robustness, yielding its highest value when all *k_i_(k_i_-1)/2* edges are present in *G_i_,* in which case a subgraph is again denoted as ideal*.* Thus, Equation. *S4a.3* provides the definition for the average *E_loc_(G):*

$E_{loc}\left(G\right)=1/N\sum_{i\in G} \frac{E\left(G_{i}\right)}{E\left(G_{i}^{ideal}\right)}$ *(Eq. S4a.3)*

Here again, the values of local efficiency range between 0 and 1.

# Section S4b

### Alternative methods for wiring cost estimation

The *Cost(G)* of building graph *G* is defined as follows:

$Cost\left(G\right)=\frac{\sum_{i\neq j\in G} A(i,j)}{\sum_{i\neq j\in G} W(i,j)}$ *(Eq. SP4b.1)*

where *A(i,j)* is the adjacency matrix (Eq.1) and *W(i,j)* is a matrix whose entries are the weights between vertices *i* and *j*, such that even if they are not connected (i.e. *a_ij_* =0 in Eq. 1), it remains possible to compute the weight of such a connection. Therefore, the *Cost(G)* function, as defined here, also accepts values between 0 and 1, following the rule for efficiency parameters. The wiring cost reaches 1 when all possible *n (n-1)/2* undirected connections are present (this assumes the higher cost of a single edge is 1). Otherwise, the cost will be defined by values lower than 1.

Prior to proceeding with the description of the weighting methods, it is fundamental to stress the difference between path lengths in weighted and unweighted graphs. For weighted graphs, the shortest path length *l_ij_* between vertices *i* and *j* is no longer the minimal number of edges to transverse between *i* and *j*, but rather the minimal sum of weights along the path between vertices *i* and *j*. Thus, it is possible that a given shortest between two vertices (see paper references 25 and 26). For the cultured neuronal networks, we opted to set *w_ij_= dij*, where *d_ij_* is the Euclidean distance between the *i^th^* and *j^th^* vertices. The longer the distance, the larger the weight assigned to this edge. This type of weighting is referred to as the ‘Euclidean’ type.

We then took into consideration the synaptic weights distribution across the network. Since the topological analysis here described studies networks features based on the properties of each of its elements, we opted to implement the disk method for the estimation of synaptic weights as explained in the *Computing vertex synaptic weight*  section in the methods). *w_ij_*, the weight of the edge connecting between the two vertices, can be assigned the inverse of the average synaptic weight around each of the two connected vertices. In this case, *w_ij_.* = 2/(*w_i_* + *w_j_*) where *w_i_* and *w_j_* are the synaptic weights measured at vertices *i* and *j*, respectively. This weighting method is referred as the ‘Marker’ method. The synaptic weight was calculated as the total fluorescence of synaptophysin-positive puncta in a disk of diameter 5 μm concentrated at each node. Selecting this radius resulted in little or no overlap between disks (not shown). Thus, an increase in total fluorescence around vertices *i* and *j* correlates with a reduction *w_ij_.*, therefore increasing the chances of selecting this edge in the shortest path across the network. To combine the morphological properties of the dendritic assembly in the network with synaptic distribution the ‘Combined’ weighting type was introduced. Here, we simultaneously considered the ‘Euclidean’ and the ‘Marker’ methods, resulting in *w_ij_* = *d_ij_*/*w’_ij_*, as previously defined. Under this joint definition, if the marker density is held constant at the vertices connected by a given edge, but the distance between vertices decreases, the corresponding edge weight will also decrease. On the other hand, if distance is held constant but the density of the synaptic marker increases, then edge weight will decrease. As previously stated in this context, a lower edge weight increases the likelihood of that edge being part of a shortest path.

While separately considering either the ‘Unweighted’ or the ‘Marker’ weighting methods, the studied networks did not meet the standards of ‘Economic’ Small-Worlds as we measured low Global efficiency values accompanied with high local Efficiency and low wiring cost (not shown). In addition, these topological networks properties were found to be synaptic-activity independent. Networks that evolved exposed to synaptic activity inhibitors attained the same topologies and were indistinguishable in terms of their efficiency and cost from the control conditions for the four weighing methods. (p>0.05 ANCOVA, n=11, 9, 9 and 9 for Ctrl, TTX, CNQX and APV, respectively).

### Section S5

### Genetic algorithm

A description of the layout of vertices’ weights that simultaneously maximized local and global network efficiencies (*E_glob_* and *E_loc_*, respectively) was obtained via a genetic algorithm (S5.1, S5.2). Here, the distribution of vertex weights (as a surrogate for synaptic weights) in two different geometrical arrangements was considered, namely those regular and aggregated arrangements that maximized the goal function (*E_glob_*+*E_loc_*)/2. Briefly, possible solutions (vertex weight distribution designs) were coded in terms of genes carried by individuals. Each gene consisted of one-dimensional vectors, where each vector element represents the weight assigned to a given vertex. Each individual has an associated fitness, i.e. the value of the goal function for the specific design. A single population of 20 individuals (i.e alternative synaptic weight configurations) bearing such genes was created and initialized to present uniformly distributed random values between 0.4 and 0.6. The core of the algorithm creates new generations by selecting high-fitness individuals as parents of the new generation, and then random mutations and recombination processes that altered the newly formed genes*.* Fitness-based selection of parents was performed via the roulette method (S5.1, S5.2). For each pair of parents, a pair of *children* individuals was created by recombining sections of a given gene from each parent, a process known as *crossover* (fraction set to 0.8)*.* A low rate of random changes *(point mutations*) was applied to elements of the children individuals (at a gene mutation level set to 0.02, uniform distribution) so as to offer the potential of creating superior children (i.e. better solutions). In addition, keeping an exact copy of the two best parents (a process known as *elitism*) improved algorithm performance. A solution was considered valid if, and only if, it was stable for a hundred generations out of a thousand maximal generations per run. A total of 25 runs were performed for each scenario and the average vertex weight across trials was reported. Since both the regular and aggregated geometric layouts have four-fold symmetry, it was possible to speed up computations by considering solely symmetric solutions, computing only the weighs of the N/4 vertices (where N is network size) that build up the top-left quarter of the lattice and assigning the same values to the vertices of the remaining lattice quarters after appropriate rotation.

**Reference**

S5.1. Goldberg DE. (1989) Genetic Algorithms in Search, Optimization and Machine Learning, Reading, Mass: Addison-Wesley Pub. Co.

S5.2. Mitchell M. (1997) An Introduction to Genetic Algorithms. Cambridge: The MIT Press.
